# Clinical features of retinal detachment treated with segmental scleral buckling

**DOI:** 10.1007/s10792-024-03186-7

**Published:** 2024-07-02

**Authors:** Meng-Chiao Wu, Yi-Yang Lee, Hsi-Kung Kuo

**Affiliations:** 1 Department of Ophthalmology, Kaohsiung Chang-Gung Memorial Hospital and Chang-Gung University, 123 Ta-Pei Road, Niao-Sung District, Kaohsiung City, Taiwan; 2 School of Medicine, Chang-Gung University, Taoyuan City, Taiwan

**Keywords:** Retinal detachment, Scleral buckling, Segmental, Pars plana vitrectomy, Surgical outcomes

## Abstract

**Purpose:**

Our study aims to evaluate the surgical outcomes and clinical features of retinal detachment (RD) cases treated with segmental scleral buckling (SB), elucidating the role of segmental SB as a vital option in specific situations during the current era.

**Methods:**

We retrospectively reviewed 128 eyes with primary rhegmatogenous RD that underwent segmental scleral buckling between November 2008 and December 2020. Clinical features and success rates were recorded and analyzed.

**Results:**

A total of 128 eyes were included. The patient’s ages ranged from 12 to 72 years, with a median age of 45. Most of the eyes were phakic (97%). Regarding the type of break, 47% were holes, and flap tears were found in 68 cases (53%). The break locations were superior-temporal (54%), inferior-temporal (31%), superior-nasal (9.5%), and inferior-nasal (5.5%). The length of the SB applied ranged from 3.5 to 8.0 clock hours, with a median of 6.0. Primary success was achieved in 121 eyes, and recurrence occurred in 7 eyes. All recurrent RD cases reattached after undergoing secondary VT. The causes of failure included 2 break reopens, 1 missed break, and 4 eyes with proliferative vitreoretinopathy. The single-surgery anatomic success (SSAS) rate for segmental SB was 94.5%. The final success rate was 100%.

**Conclusions:**

For phakic, low complexity retinal detachment in our study, segmental scleral buckling emerges as a surgical option with a high primary success rate and a lower incidence of complications.

## Introduction

As the incidence of rhegmatogenous retinal detachment (rhegmatogenous RD) increases at a rate of 1.7 ± 0.1 cases per 100,000 person-years per year, the surgical The management of rhegmatogenous retinal detachment (RD) remains one of the most challenging conditions encountered in retinal clinical practice [1]. Surgical management includes pneumatic retinopexy, scleral buckling (SB), pars plana vitrectomy (VT), or VT combined with SB (VT/SB). While the technique of vitrectomy has become more advanced, a significant decline in primary SB usage, from 84 to 5–7% within the last 1–2 decades, has been reported[2–4]. Despite the decreasing prevalence of SB, previous studies have reported some benefits. In carefully selected cases, SB has shown a higher single operation success rate compared to VT [5]. For patients with uncomplicated retinal detachment, SB management remains a good option due to the lower final failure rate in retinal detachment repair compared to VT, with or without a supplemental buckle [6]. Segmental SB is a variant type of SB that provides localized support to specific areas of the retina where detachment or breaks are present. This minimally invasive surgical procedure is another choice for uncomplicated retinal detachment, as there is no statistically significant difference in the failure rate compared to encircling buckles [6]. Furthermore, segmental SB has shown a minimum of postoperative complications in previous studies with long-term follow-up [7]. Based on the outcomes and patient characteristics discussed in a few studies involving rhegmatogenous RD cases treated with segmental SB, this study was conducted to investigate the clinical features of retinal detachment treated with segmental scleral buckling.

## Methods

### Study design and eligibility

This was a retrospective, cohort study conducted at Kaohsiung Chang-Gung Memorial Hospital, a single tertiary medical center in Southern Taiwan. The study included eyes that underwent operations for primary rhegmatogenous RD between November 2008 and December 2020. The inclusion criteria encompassed treatment-naïve patients (defined as those without prior surgical interventions such as pneumatic retinopexy, scleral buckling, or vitrectomy), preoperative clear ocular media, the use of segmental scleral buckling as the surgical method, and a postoperative follow-up period longer than 3 months. Exclusion criteria consisted of cases involving penetrating trauma, the presence of proliferative vitreoretinopathy, syndromic retinal detachment, coexisting choroidal detachment or macular hole, a history of previous complicated intraocular surgery, glaucoma, or other ocular diseases (e.g., diabetic retinopathy, occlusive vasculopathy, uveitis, macular pucker, myopic tractional maculopathy). The approach to patient selection for segmental scleral buckling included cases with breaks within one quadrant or 90°, regardless of the number, type (tear or hole), or location of the break. Patients with multiple breaks exceeding 90° circumferentially required encircling scleral buckling in our clinical practice. Furthermore, encircling scleral buckling was performed for cases with proliferative vitreoretinopathy. The extent of retinal detachment, the presence of lattice degeneration, macula-on/off status, and the presence of high myopia did not preclude the decision to perform segmental scleral buckling. Cryopexy was routinely applied during the segmental SB operation to address lattice degeneration, irrespective of its location. The patient selection mentioned above introduce the concept of low complexity retinal detachment in our study, which, in contrast, is distinguished from the definition of “moderately complex” determined by PRO study [8].

Between November 2008 and December 2020, a total of 128 primary rhegmatogenous RD patients who underwent surgery with segmental SB, performed by a single experienced retinal surgeon, HKK, were enrolled in the study. This study received ethical approval from the Chang Gung Memorial Hospital Institutional Review Board (IRB number 202301861B0D001) and written informed consent was not required under the decision of Chang Gung Memorial Hospital Institutional Review Board.

### Surgical techniques

**Fig. 1:**
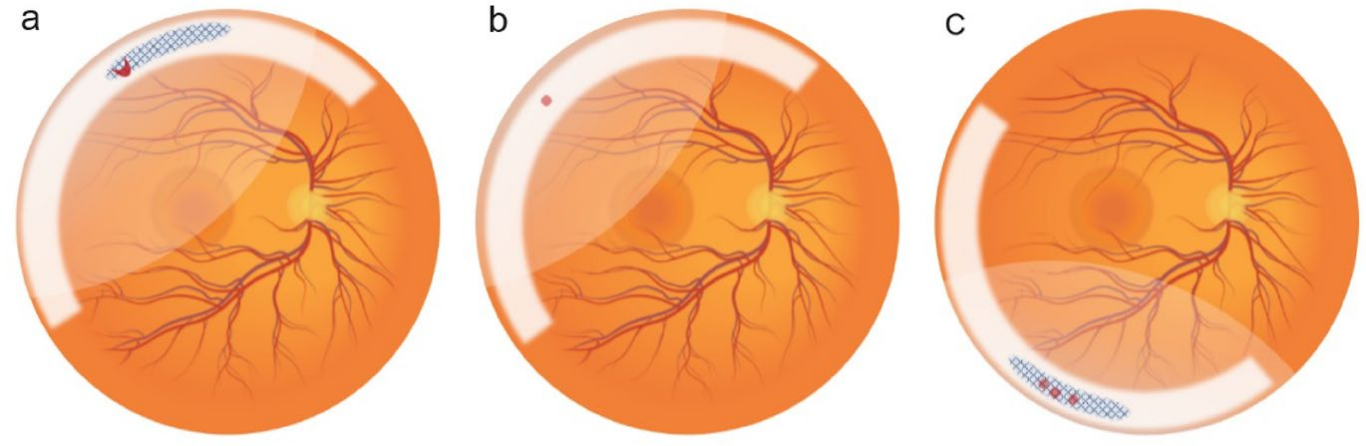
A schematic diagram of circumferential segmental buckling under different types of rhegmatogenous retinal detachment (RRD). **a** Macula-off RRD with a lattice and a flap tear. **b** Macula impending-off RRD with a hole. **c** Macula-on RRD with lattices and holes

In Dr. Kuo’s buckling procedures, silicone sponge (#506, MIRA Inc) was placed for circumferential segmental buckling across at least 2 rectus muscles (Fig. 1) followed with cryopexy around the break. External subretinal fluid drainage and gas tamponade were performed at the surgeon’s discretion.

### Data collection and outcome assessments

Data, including patient demographics, ocular history, characteristics of RD, and details of surgical intervention, were collected. The characteristics of RD encompassed macula status, extent of RD, type of retinal break, number of retinal breaks, and the location of retinal breaks. Types of retinal breaks were categorized as flap tears or atrophic holes. Eyes with both flap tears and holes were classified as retinal tears. Several consecutive small atrophic holes at the same lattice degeneration were considered as a single hole. We also classified the locations of breaks as superior temporal, inferior temporal, superior nasal, inferior nasal, and multi-quadrant areas. Features of surgical interventions included the length of the segmental SB, the use of external subretinal fluid drainage, and C3F8 gas tamponade.

The primary outcome was assessed based on the anatomically successful reattachment of the retina after a single operation without any other interventions within 90 days, denoted as single surgery anatomic success (SSAS). Secondary operations, including any subsequent vitreoretinal surgeries during the follow-up period, were also recorded. The final anatomically successful reattachment was recorded, regardless of the use of silicone oil after vitrectomy.

### Statistical analysis

Statistical analysis was conducted using IBM SPSS Statistics for Windows, version 25 (IBM Corp., Armonk, N.Y., USA). Baseline characteristics were presented as mean ± SD for continuous parameters and as counts and percentages for categorical variables. To compare the factors associated with SSAS, patients were divided into two groups: the SSAS group and the non-SSAS group. Continuous variables were compared using an unpaired t-test, while categorical variables were analyzed using either the chi-squared test or Fisher’s exact test, as appropriate. Subgroup analysis was performed to assess the preference for gas tamponade in addition to segmental scleral buckling.

## Results

A total of 128 eyes from 128 patients (56 females, 43.8%), with a mean age of 46.3 ± 15.3 years (range: 12–76 years), were included in our study. Basic patient characteristics and features of retinal detachment are summarized in Table 1. Most patients (*n* = 125, 97.7%) were phakic at baseline, with a mean follow-up time of 29.6 ± 25.8 months (range: 3–147 months) after surgery. Overall, retinal flap tears were found in 68 cases (53.1%), with a mean number of 1.42 ± 0.66 breaks (range: 1–4 breaks). More cases had breaks in the superior hemisphere (*n* = 88, 68.8%) than in the inferior hemisphere, with the majority located in the superior temporal quadrant (*n* = 70, 54.7%). The extent of retinal detachment averaged 4.25 ± 1.70 clock hours (range: 2–12 clock hours). Among these 128 eyes, 55 out of 128 (43%) were macula-on, while the other 73 eyes were macula-off (57%).

**Table 1:** Basic characteristics of the patients and retinal detachment

	**Overall (*n* = 128)**
Age (years), mean ± SD (median, range)	46.3 ± 15.3 (51, 12–76)
Female, *n* (%)	56 (43.8%)
Phakic, *n* (%)	125 (97.7%)
Follow-up time (months), mean ± SD (median, range)	29.6 ± 25.8 (23.5, 3–147)
Features of retinal detachment	
Retinal flap tear/hole, *n* (%)	68/60 (53.1%/46.9%)
No. of breaks, mean ± SD (median, range)	1.42 ± 0.66 (1, 1–4)
Break located in superior hemisphere, *n* (%)	88 (68.8%)
Break located in ST quadrant, *n* (%)	70 (54.7%)
RD extent (clock hours), mean ± SD (median, range)	4.25 ± 1.70 (4, 2–12)

Characteristics of management with segmental SB and outcomes are presented in Table 2. Segmental SB had an average length of 5.65 ± 0.99 clock hours (range: 3.5–9.0 clock hours). External subretinal fluid drainage was performed in 112 cases (87.5%), while C3F8 tamponade was only employed in 44 cases (34.4%). Overall, 121 patients (94.5%) achieved single surgery anatomic success (SSAS), while 7 cases failed (5.46%). The causes of failure included 2 reopened breaks, 1 missed break, and 4 eyes with proliferative vitreoretinopathy (PVR). These 7 failed cases underwent consecutive vitrectomy and all achieved reattachment. Additionally, 2 initially attached cases required vitrectomy for macular pucker and new RD during the follow-up period. In total, 9 eyes underwent secondary operations.

**Table 2:** Characteristics of management with segmental SB and outcomes

	Overall (*n* = 128)
Characteristics of management	
Length of segmental SB (clock hours), *mean* ± *SD (median, range)*	5.65 ± 0.99 (5.5, 3.5–9.0)
External drainage,* n (%)*	112 (87.5%)
Gas tamponade,* n (%)*	44 (34.4%)
Outcomes	
Single surgery anatomic success^*^,* n (%)*	121 (94.5%)
Secondary operation^**^,* n (%)*	9 (7.03%)
Final re-attachment,* n (%)*	128 (100.0%)

^*^Single surgery anatomic success is defined as retinal attachment with no additional RRD surgery within a 90-day period

^**^Secondary operation includes any subsequent vitreoretinal surgery during the follow-up period

There were 86 patients with phakic eyes, a follow-up duration of greater than or equal to 12 months, and no vitrectomy after segmental scleral buckling. Among these 86 patients, 8 underwent cataract surgery afterward, representing a percentage of 9.3%. The average age at which RD occurred was 62.2 years old, and the average interval between prior segmental scleral buckling and cataract surgery was 3.16 years (range: 0.5–6.3 years).

The comparison between the SSAS group and the non-SSAS group is presented in Table 3. There were no significant differences in age, gender, lens status, number and location of retinal breaks, length of SB, and the use of external drainage between the failure group and the SSAS group. A tendency toward the presence of flap tears instead of atrophic holes was observed in the non-SSAS group but did not reach statistical significance. However, the SSAS group had a smaller extent of retinal detachment (4.11 ± 1.51 clock hours vs. 6.57 ± 3.05 clock hours), and there was a significantly higher percentage of gas tamponade use in the non-SSAS group. To analyze the factors influencing the use of gas tamponade, we compared basic characteristics and clinical features of retinal detachment, revealing that patients older than 50 years old, with flap tears, superior breaks, and more extensive retinal detachment were all associated factors **(**Table 4**)**.

**Table 3:** Factors associated with single surgery anatomic success

	Single surgery anatomic success		*p* value
	Yes (*n* = 121)	No (*n* = 7)	
Age (years), *mean* ± *SD (median, range)*	45.9 ± 15.1 (51, 12–73)	53.0 ± 17.6 (54, 32–76)	0.234
Age ≤ 50 years,* n (n/n of subgroup, %)*	60/63 (95.2%)	61/65 (93.8%)	0.729
Female,* n (%)*	52 (43.0%)	4 (57.1%)	0.463
Phakic,* n (%)*	118 (97.5%)	7 (100.0%)	0.802
Retinal flap tear,* n (%)*	62 (51.2%)	6 (85.7%)	0.076
No. of breaks, *mean* ± *SD (median, range)*	1.43 ± 0.67 (1, 1–4)	1.29 ± 0.49 (1, 1–2)	0.576
Break located in superior hemisphere,* n (%)*	82 (67.8%)	6 (85.7%)	0.433
Break located in ST quadrant,* n (%)*	66 (54.5%)	4 (57.1%)	0.893
RD extent (clock hours), *mean* ± *SD (median, range)*	4.11 ± 1.51 (4, 2–10)	6.57 ± 3.05 (5, 3–12)	< 0.001
SB length (clock hours), *mean* ± *SD (median, range)*	5.66 ± 1.00 (5.5, 3.5–9.0)	5.50 ± 0.96 (6, 3.5–6.0)	0.686
External drainage,* n (%)*	105 (86.8%)	7 (100.0%)	0.595
Gas tamponade,* n (%)*	39 (32.2%)	5 (71.4%)	0.047

**Table 4:** Factors associated with preference of gas tamponade

	Gas tamponade		*p* value
	Yes (*n* = 44)	No (*n* = 84)	
Age > 50 years,* n (n/n of subgroup, %)*	34 (77.3%)	31 (36.9%)	< 0.001
Phakic,* n (%)*	44 (100.0%)	81 (96.4%)	0.551
Retinal flap tear,* n (%)*	39 (88.6%)	29 (34.5%)	< 0.001
Break located in superior hemisphere,* n (%)*	43 (97.7%)	45 (53.6%)	< 0.001
RD extent (clock hours), *mean* ± *SD (median, range)*	4.89 ± 2.41 (4, 2–12)	3.91 ± 1.04 (4, 2–7)	0.002

## Discussion

The choice between scleral buckling and vitrectomy depends on various factors, including the number, type, and location of retinal breaks, the extent of the detachment, the presence of proliferative vitreoretinopathy, and the surgeon’s expertise and preferences. Offering a better visual and anatomic outcome, SB was preferred for uncomplicated rhegmatogenous RD cases, characterized by features such as a single break or breaks within one quadrant, phakic lens status, and absence of PVR grade C. Conversely, VT was reserved for complex cases with pseudophakic or aphakic lens status, especially when rhegmatogenous RD was anticipated to have a poor response to external approaches. These complex cases included multiple breaks, giant retinal tears, bullous detachments and PVR grade C. A combined approach of VT and SB (VT/SB) was chosen in patients with failed prior surgical intervention, inferior break detachments and other complex situations, yielding better anatomic outcomes [9–12]. With advancements in micro-incision vitrectomy surgery (MIVS) and smaller gauge instrumentation, along with the influence of surgeon experiences, VT has become the preferred and prevailing approach for uncomplicated rhegmatogenous RD in the current era. The benefits that contribute to the popularity of VT over SB include shorter operating times and improved intraoperative uncomfortable experiences for patients [13]. Similarly, multiple studies have shown a trend of increasing VT/SB ratios. For instance, a study conducted by Sedova et al. [14] revealed a continuous decrease in scleral buckling surgeries from 95 to 16%, accompanied by a significant increase in vitrectomies during the years 2004–2012. In a similar vein, Madi and Keller [4] reported that pars plana vitrectomies, regardless of the indication, increased fourfold from 5761 to 26,900 procedures, while scleral buckling surgeries decreased by two-thirds from 2897 to 780 cases between 2000 and 2018 in England. Additionally, the smaller gauge instrumentation (such as 23- or 25-gauge) is reported non-inferior to traditional 20-gauge vitrectomy [15, 16]. Thus, MIVS has become increasingly adopted by ophthalmic surgeons worldwide, leading to a notable rise in the number of vitrectomies being performed annually.

Vitrectomy is highly effective in treating complex retinal detachment cases. The Scleral Buckling versus Primary Vitrectomy in Rhegmatogenous Retinal Detachment (SPR) study, published in 2007, was a pivotal milestone that provided vitreoretinal surgeons with clear guidance for managing medium-complexity rhegmatogenous RD cases. According to the study’s results, in the phakic eye subgroup, scleral buckling offers significant advantages over vitrectomy in several aspects. These benefits include better best-corrected visual acuity (BCVA) improvement, delayed progression of cataract formation, and no inferiority in anatomical outcomes or postoperative PVR rates. Conversely, in the aphakic/pseudophakic subgroup, VT offers advantages in terms of a higher primary anatomical success rate and a lower rate of reoperations affecting the retina. However, there is no statistically significant difference in BCVA improvement or post-operative PVR rates. Based on these varying clinical outcomes, it is recommended to consider scleral buckling for medium-complexity rhegmatogenous RD in phakic eyes, while vitrectomy is recommended for aphakic/pseudophakic eyes [17]. Another influential study, the Primary Retinal Detachment Outcomes Study Report Number 2, carried out by Ryan et al., where only phakic eyes were included, showed the similar outcome with SPR study. Among phakic patient with medium complexity rhegmatogenous RD, SB whether combined with VT or not, had superior single surgery anatomical success rated than VT alone. However, the study found that patients who underwent VT alone or VT combined with SB (VT/SB) had a significantly higher percentage to receive subsequent cataract surgery compared to those who underwent SB alone [8].

A comprehensive meta-analysis, which included multiple studies involving 15,947 eyes, revealed that SB had a higher incidence of choroidal/subretinal hemorrhage, choroidal detachment, and residual sub-retinal fluid compared to VT. Interestingly, the primary and final reattachment rates were found to be similar between VT and SB [18]. Regarding complications, patients who underwent VT had a higher incidence of postoperative cystoid macular edema compared to those treated with SB. Additionally, the development of epiretinal membrane was found to be correlated with older age, regardless of the surgical procedure [19]. In summary, both vitrectomy and scleral buckling have their unique advantages and associated risks. The selection of the surgical approach should be made thoughtfully, taking into consideration the specific characteristics of the retinal detachment and individual patient factors.

The encircling SB technique involves positioning a silicone band around the entire circumference of the eye, providing broad support for retinal detachment, particularly in cases of extensive detachment or multiple breaks. Nevertheless, its application can be linked to an increased risk of postoperative complications, such as significant myopic changes, extraocular muscle imbalance, and scleral erosion [20]. On the other hand, segmental scleral buckling (segmental SB) offers a more localized approach, which reduces the likelihood of encountering such complications. Segmental SB offers localized support to the detached retina by gently indenting the sclera at the site of the retinal break. This indentation acts as a mechanical barrier, preventing the flow of subretinal fluid and facilitating reattachment. It’s worth noting that in uncomplicated phakic retinal detachments, the European Vitreoretinal Society Retinal Detachment Study found no statistically significant difference in failure rates between segmental and encircling buckles [6]. Common complications linked to segmental SB include issues such as SB extrusion, bleeding, infection, diplopia, and astigmatism induced by the buckle [21]. In a previous study, Kreissig et al. [22] reported PVR grade C rate of 1.9%, missed breaks in 0.8% of cases, and exposure or infection of the sponge buckle in less than 0.5% of patients receiving segmental SB without drainage. The low rate of PVR in our study might be due to the exclusion of patients with preoperative PVR, which accounted for 2.9% in Kreissig’s review. Additionally, the shorter follow-up period in our study might have underestimated the complication rate related to buckle materials.

Despite cataract-related issues being more manageable today, considerations such as visual impairment due to cataract formation, the cost and risks associated with subsequent cataract surgery, and the loss of accommodation and parallax following intraocular lens insertion remain important factors to bear in mind, especially for younger patients with clear lens status.

In our study, the average interval between prior segmental scleral buckling and cataract surgery was 3.16 years. This stands in contrast to previous studies in which patients underwent vitrectomy [23, 24], the segmental SB procedure may be associated with a delay in cataract surgery. Compared to VT, SB led to the lowest rate of cataract formation and progression [25] A possible mechanism for this delay could be the avoidance of increased intraocular oxygen tension via reduced intraocular manipulation, with preservation of the anterior vitreous face and the avoidance of vitreous substitutes (e.g., gas, silicone oil) [26].

Pneumatic retinopexy is another option for the treatment of uncomplicated rhegmatogenous RD. It offers several advantages, including being less invasive, having a shorter operative time, lower cost, and quicker visual recovery. Additionally, pneumatic retinopexy can be performed in outpatient clinics, making it accessible in less developed areas. However, its success depends heavily on proper patient positioning, which may limit its suitability for some individuals. In a retrospective cohort study by Yannuzzi et al. [27], a SSAS rate of 68.5% was reported in eyes with noncomplex rhegmatogenous RD treated with primary pneumatic retinopexy. A comparative study conducted by Paulus et al. [28] compared pneumatic retinopexy to SB and reported that SB had a significantly higher single surgery reattachment rate than pneumatic retinopexy, along with better final visual acuity (VA) improvement. Furthermore, a Cochrane review compared pneumatic retinopexy with SB for repairing simple rhegmatogenous RDs and found that pneumatic retinopexy had a slightly lower retinal reattachment rate and a higher risk of recurrent retinal detachment [29]. During the same period of our study, the author only performed pneumatic retinopexy for a few cases.

In our study, we included 128 eyes, with the majority being phakic (97.7%). The median length of the SB applied was 5.65 clock hours. The SSAS rate for segmental SB was 94.5%, and the final success rate was 100%. Among the 7 recurrent RD cases, all were successfully reattached after undergoing secondary vitrectomy (VT). According to the review of 1462 detachments with segmental SB without drainage, Kreissig et al. [22] reported the primary attachment rate of 91% and final attachment rate of 97%. The difference in the primary attachment rate might have resulted from more stringent patient selection and different demographics in our study. In contrast to the aphakic rate of 19.6%, pseudophakic rate of 0.9%, and vitreous hemorrhage rate of 15.9% in Kreissig’s previous study, our study only had 2.3% pseudophakic eyes with no aphakic eyes or patients with vitreous hemorrhage [7]. According to the results of the pseudophakic/aphakic trial in SPR study, encircling SB alone had the lowest success rate compared to VT or VT/SB. The possible explanation was that the aphakic lens status implies a more complex RD condition, such as stronger vitreous traction or an increased risk of PVR development [30]. Additionally, the exclusion of patients with preoperative PVR mentioned above contributed to the lower failure and PVR rates.

There are several limitations in our study. Being a retrospective case series, one inherent limitation is the absence of a comparison group to determine potential differences among other intervention types and identify any outstanding approaches. The lack of a control group prevents us from establishing definitive outcome differences between surgical techniques such as vitrectomy or encircling SB. Additionally, there may be biases related to outcomes, including the complexity of retinal detachment cases, patient’s underlying medical conditions, and individual surgeon preferences. Furthermore, the study population spanning 12 years potentially introduces bias due to learning effects and advancements in equipment over time. However, a notable strength of this study is that all segmental SB procedures were performed by the same surgeon, which minimizes bias associated with operator factors. While acknowledging the limitation of not having a comparison group, this study still provides valuable real-world data on the clinical outcomes and safety profile of segmental SB in a large patient cohort treated over an extended period. Despite its retrospective nature, the consistent surgical approach and the sizeable cohort contribute meaningful insights into the efficacy and role of segmental SB in the management of rhegmatogenous RD.

## Conclusions

For phakic, low complexity retinal detachment in our study, segmental scleral buckling emerges as a surgical option with a high primary success rate and a lower incidence of complications.

## Data Availability

The datasets used and/or analyzed during the current study are available from the corresponding author on reasonable request.
